# Dislodged biliary stent causes lower gastrointestinal hemorrhage four years postendoscopic retrograde cholangiopancreatography

**DOI:** 10.1002/ccr3.1585

**Published:** 2018-05-15

**Authors:** Ioannis S. Papanikolaou, Georgios Tziatzios, Paraskevas Gkolfakis, Stavros Parasyris, Polyxeni Kizgala, Nikolaos Economopoulos, Iordanis N. Papadopoulos, George D. Dimitriadis, Konstantinos Triantafyllou

**Affiliations:** ^1^ Hepatogastroenterology Unit Second Department of Internal Medicine—Propaedeutic Research Institute and Diabetes Center Athens Greece; ^2^ 4th Department of Surgery Medical School National and Kapodistrian University of Athens “Attikon” University General Hospital Athens Greece; ^3^ 2nd Department of Radiology Medical School National and Kapodistrian University of Athens “Attikon” University General Hospital Athens Greece

**Keywords:** biliary, bleeding, colon, stent

## Abstract

Endoscopic biliary stent placement is an efficient method for the decompression of the biliary system in various benign and malignant causes. Dislocation and stent migration is a well‐known complication, with most displaced stents passing through the bowel, uneventfully. Rarely, migrated stents can be accounted for potentially life‐threatening complications.

## 1 QUESTION

**Figure 1:**
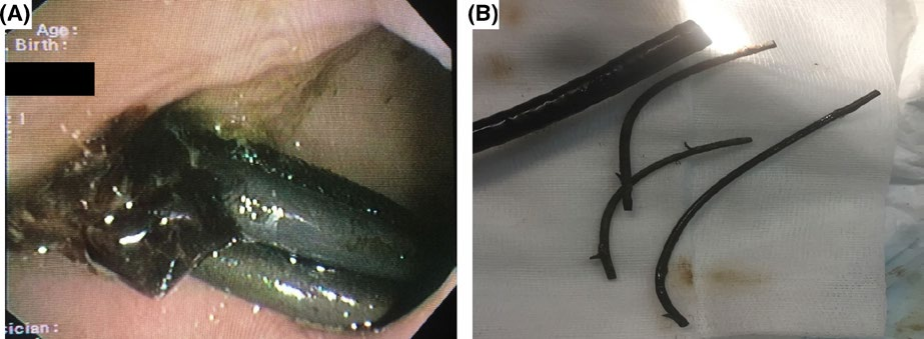
A, Endoscopic view showing protrusion of the distal part of the stents with adherent clot. B, Two straight plastic biliary stents 12 cm, 10 Fr and one 7 cm, 10 Fr successfully removed

**Figure 2:**
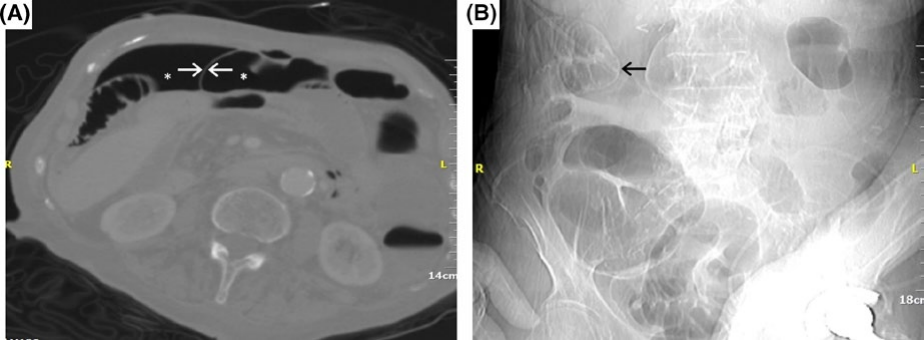
A, Abdomen computed tomography (IV contrast lung window). Free abdominal gas (White stars) and falciform ligament (White arrows). B, Plain abdominal radiograph. Clear delineation of small bowel wall at right upper quadrant, due to free intraabdominal gas (Black arrow)

**Figure 3:**
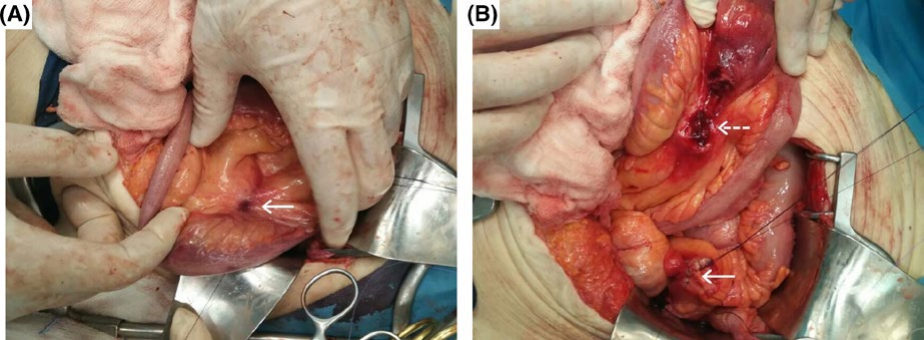
A, First sign of localized peroration, recognized as a defect in the mesentery of a small bowel loop (Arrow). B, The site of sigmoid perforation (Arrow) and press ulcer in the mesentery of the small bowel (Dotted Arrow)

A 87‐year‐old patient was referred due to hematochezia. Four years ago, she underwent endoscopic retrograde cholangiopancreatography (ERCP) with 3 straight plastic biliary stents' insertion, but she was lost to further follow‐up. Colonoscopy identified the stents impacted in a sigmoid colon diverticulum with adherent blood clot (Figure 1A). They were retrieved using biopsy forceps (Figure 1B), revealing a mucosal defect. Extreme bowel angulation precluded endoclips' use. A few hours later, the patient deteriorated; computed tomography revealed free abdominal gas (Figure 2A,B); she was transferred to the operating room. A mesentery defect of the small intestine and an imprint of the sigmoid in the right‐lateral pelvic wall were detected (Figure 3A,B). Partial colectomy of the sigmoid, closure of the anorectal stump, end‐colostomy and a suture of the mesenteric mesh of the small intestine was performed.

## 2 DIAGNOSIS

### 2.1 A migrated biliary stent lodged within a colonic diverticulum

Time to plastic biliary stents migration ranges from 2 weeks to 3 years post‐ERCP.^1^ Stent colonic impaction may arise due to diverticular disease, while hemorrhage results from stent‐induced mucosal injury.^2^ We postulate that the migrated stent became lodged within a diverticulum and its distal end eroded the antimesenteric border resulting in gastrointestinal bleeding. Perforation occurred after stent removal.

## INFORMED CONSENT

Written informed consent was obtained.

## CONFLICT OF INTEREST

None declared.

## AUTHORSHIP

ISP: revised the draft and approved the manuscript. PG, GT, SP, and PK: drafted and approved the manuscript. NE, INP, GDD, and KT: conceived the idea, revised the draft and approved the manuscript.
